# Recovering from trampling: The role of dauciform roots to functional traits response of *Carex filispica* in alpine meadow

**DOI:** 10.1002/ece3.10709

**Published:** 2023-11-03

**Authors:** Rong Fan, Wanting Liu, Songlin Jiang, Yulin Huang, Wenli Ji

**Affiliations:** ^1^ College of Landscape Architecture and Arts Northwest A&F University Yangling China

**Keywords:** alpine meadow, functional traits, plant economic spectrum, root system, trampling

## Abstract

In the natural habitats of China, dauciform roots were only described in degraded alpine meadows. It was found that the presence of dauciform roots of *Carex filispica* was related to the advantage of multiple functional traits after trampling, reflecting short‐term resistance. However, the long‐term response of dauciform roots to trampling and the recovery of *C. filispica* with and without dauciform roots to trampling require further studies. In this study, different intensities of trampling (0, 50, 200 and 500 passages) were performed in an alpine meadow. One year later, individuals with and without dauciform roots were separated and their functional traits related to the economic spectrum of leaves and roots were measured as a reflection of recovery from trampling. The results showed that: (1) 1 year after trampling, the number of dauciform roots showed an increase with trampling intensity; (2) 1 year later, there was no significant difference in the response of economic spectrum traits among trampling intensities, or between plants with and without dauciform roots; (3) the number of dauciform roots was positively correlated with the leaf area of both individuals with and without dauciform roots, as well as with the biomass of those without dauciform roots; and (4) plants with more resource‐conservative roots showed an advantage after trampling recovery: specifically, plants with dauciform roots showed such an advantage in the control group, which was lost with a leaning towards resource‐acquisitive roots and an increased density of dauciform roots once trampled. In contrast, plants without dauciform roots showed a significant advantage of conservative roots only after trampling. In conclusion, the presence of dauciform roots is related to the plants' position on the root economic spectrum, thereby influencing the recovery of *C. filispica* from trampling. *Carex filispica* showed strong recovery from trampling after 1 year, which makes it an adequate choice for ecological restoration in alpine meadows. Dauciform roots showed a positive correlation with the aboveground growth of both plants with and without them, however, it requires a lab‐controlled study to confirm whether there is indeed a positive effect on the growth of neighbouring plants.

## Graphical abstract

The number of DRs increased with trampling intensity after a long‐term recovery, which has a positive relationship with the aboveground growth of both individuals with and without DRs. The presence of DRs may be related to the plants' position on the economic spectrum, thus influencing the resilience of *Carex filispica* to trampling.

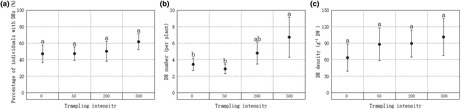

## 1 INTRODUCTION

Currently, the majority of meadows in China are suffering from various stages of degradation due to climate change and human disturbance (Wang et al., 2015). As the major human disturbance, the trampling of overgrazing and tourism have been proven to have a dramatic effect on meadow degradation at a small scale (Ballantyne et al., 2014; Dong et al., 2013; Henn et al., 2018; Liu et al., 2018; Zhou et al., 2018). While trampling has a direct damaging effect on the aboveground parts of plants, it also affects the belowground parts indirectly by the modifications to soil physical properties and nitrogen mineralization (Breland & Hansen, 1996; Głąb, 2014; Wang et al., 2020). To state the response of plants to trampling, one must combine resistance, resilience and tolerance (Gallet & Roze, 2002), which were traditionally calculated by the total plant cover variation, while it was previously stated that indices solely based on cover do not seem to be a good indicator for defining pressure thresholds (Gallet & Roze, 2002). In the meantime, variation in functional traits of plants is proved to be strongly associated with their response to the disturbances, while more than 50% of the variation in resistance, resilience and tolerance can be explained by morphological variation (Cole, 1995): the short‐term response reflects resistance and the long‐term response reflects tolerance and resilience (Liddle, 1975; Roovers et al., 2004), which were not necessarily determined by the same characteristics (Cole, 1995). In recent years, using functional traits to predict the coping strategies of plants to human and natural disturbances has been well attended (Laliberte et al., 2010; Pakeman, 2011), yet studies on the response to human trampling are still limited (Bernhardt‐Römermann et al., 2011).

The functional traits of plants can reflect their response to the environments (Watson & Szathmáry, 2016), of which leaf traits were adequately studied and appeared to be in a global pattern: in 2004, the leaf economic spectrum (LES) was proposed, summarizing the patterns of variation and correlation of leaf traits at a global scale (Reich, 2014; Wright et al., 2007; Wright & Westoby, 2003). On the one hand, the plants' position on the leaf economic spectrum was proved to be significantly related to their response to disturbances (Oram et al., 2020). On the other hand, roots, with diverse variations and great plasticity which are highly influenced by the environment (Freschet et al., 2017; Larson & Funk, 2016), have not yet been adequately or consistently studied (Reich, 2014). Furthermore, the presence of special root structures has made the situation more complicated (Lambers et al., 2008): apart from the classical fast–slow conservation gradient, their collaboration with the regular root system, ranging from “do‐it‐yourself” to “outsourcing” of resource uptake, also need to be taken into consideration in the conceptual framework of root economics (Bergmann et al., 2020).

Dauciform root (DR), which can dissolve the soil‐fixed phosphorus (P) through carboxylate exudation, is a special type of root mostly found in Cyperaceae (Lambers et al., 2019; Shane et al., 2005). Cyperaceae plays a key role in the ecological stability of alpine meadows (Yang et al., 2023) and shows a unique performance in degraded alpine meadows of China: their biomass and leaf area remained stable with the increasing degradation while grasses and legumes showed a significant decrease (Hao et al., 2020; Ma et al., 2010), which leads to a gradual replacement under advanced stages of degradation (Lin et al., 2015). These degraded alpine meadows, in the meanwhile, are the only natural habitats where dauciform roots were described in China (Gao & Yang, 2010), which begs the question: is there a link between the unique performance of Cyperaceae and the presence of dauciform roots? As a matter of fact, as a responding strategy of plants, dauciform roots are often found in disturbed habitats (Brundrett, 2009) and proved to be related to the response of *Carex filispica* to trampling: 2 weeks after trampling, the proportion of individuals with DRs was found to be positively related to the leaf size in total, while individuals with DRs had more aboveground biomass than those without DRs after mild and moderate trampling (Fan et al., 2023). However, the relationship between dauciform roots and the functional traits of plants in long‐term recovery from trampling has not yet been clarified (Fan et al., 2023), while recovery is an essential combination of resistance and resilience and must be taken into consideration (Gallet & Roze, 2002).

Based on the background story, we proposed these hypotheses: A. The properties of dauciform roots have a different long‐term response to trampling from the short‐term one. B. Individuals with and without DRs showed different responses in functional traits to trampling after 1 year. C. The presence of dauciform roots is beneficial to the recovery of *C. filispica* species from trampling. To test these hypotheses and explore the relationship between dauciform roots and long‐term recovery from trampling, an alpine meadow dominated by *C. filispica* at Baima Snow Mountain was selected and a quantitative trampling experiment was conducted. This study aimed to explore the ecological adaptability of *C. filispica*, revealing the mechanism by which it becomes a dominant species in degraded alpine meadows, laying a foundation for further research on dauciform roots and providing a basic information and scientific basis for the proper restoration and reconstruction of degraded alpine meadow ecosystems.

## 2 METHODS

### 2.1 Study area

This study is located at Baima Snow Mountain Pass (4300 m, 28°20′9″ N, 99°4′36″ E) in Baima Snow Mountain National Nature Reserve, Deqin County, Yunnan Province. This area has a cold‐temperate mountain monsoon climate with a mean annual temperature of −1.0°C and precipitation of 600–650 mm (Gao & Yang, 2016). The site selected for the trampling experiment is mainly an alpine scrub–meadow zone, dominated by *C. filispica* and *Polygonum viviparum*, which is exposed to a moderate degree of grazing, as well as tourism activities: it is a tourist spot due to the convenience of National Highway 214 passing by.

### 2.2 Experimental design

In September 2021, a relatively flat area of homogeneously distributed vegetation with similar community composition was chosen and six replicate sets of experimental trampling lanes were established. Each set comprised four lanes, each 2 m long and 0.5 m wide, and each two were separated by 1 m. Treatments were performed in the same order (lowest‐to‐highest trampling intensity) in each set, while the sets were not placed in a line to avoid positional effects and separated from each other by at least 1 m to avoid interaction effects. The experimental trampling was applied based on standard experimental procedures recommended by Cole (Cole & Bayfield, 1993). One lane was a control with no trampling received, the other three lanes received three trampling treatments of different intensities (50, 200 and 500 passages, a one‐way trip along the lane is counted as one pass), which were performed in 1 day after a period of rain (Bayfield, 1979). These trampling intensities were selected as previous studies in trampling disturbance had found that these levels are the indicating thresholds of plant tolerance (Cole & Bayfield, 1993; Gallet & Roze, 2002), while a tolerance of 500 passages indicated highly tolerant, 200 characterized moderately tolerant and 50 was chosen to represent highly sensitive vegetation. Three participants with similar weights of about 90 kg each performed in two replicate sets. The chosen area was previously exposed to grazing and sightseeing, and after the treatments, the site was surrounded by marked ropes to avoid further trampling.

### 2.3 Trait measurements

One year after the trampling (September 2022), the plants and surrounding soil plot of 10 × 10 × 10 cm were collected from each lane, and the *C. filispica* individuals containing nearly the entire root system were separated, washed off the soil and sorted by whether they have DRs. After which, multiple functional traits were measured to reflect their recovery from trampling: Leaf area (LA), specific leaf area (SLA), leaf dry matter content (LDMC) and leaf nitrogen (N) content were selected for the leaf economic spectrum (LES) traits, while root area (RA), specific root area (SRA), root density (RTD) and root nitrogen (N) content were selected for the root economic spectrum (RES) traits. Previous studies showed that plants with smaller leaf area (LA) tended to have higher resistance towards trampling; specific leaf area (SLA) reflects the balance of investment in growth and defence, which tends to have a positive relation with trampling resilience; lower leaf dry matter content (LDMC) and higher leaf N content are associated with faster growth (Bernhardt‐Römermann et al., 2011; Cornelissen et al., 2003). While root traits were not commonly tested in previous studies of trampling, they reflect the response to the variation of soil properties and can affect the status of aboveground growth. Root area (RA) is expected to be limited under high trampling, while higher specific root area (SRA) and lower root density (RTD) showed a tendency to assure fast resource acquisition, leading to high N content in the fine roots (Bloom et al., 1985; Reich, 2014).

The proportion of individuals with DRs was recorded, and the average number and density (total number of DRs/root dry weight) of DRs per individual were calculated using an XTL‐3B stereoscopic microscope. All the leaves of three individuals with DRs and three without DRs in each lane were removed and measured by LI‐COR portable leaf area meter to determine the total leaf area of each plant. The whole root systems of these individuals were then scattered in the water carefully without overlapping, and their pictures were scanned and analysed by LA‐S root analyser to determine the root surface area of each plant. Afterwards, their above‐ and belowground parts were separately dried to constant weight and weighed for biomass. The nitrogen content was determined by the Indophenol blue colorimetric method after digestion with H_2_SO_4_‐H_2_O_2_ (Bao, 2000). The specific leaf area (leaf area/leaf dry weight), leaf dry matter content (leaf dry weight/leaf fresh weight), specific root area (root surface area/root dry weight) and root density (root dry weight/root volume) were calculated.

### 2.4 Statistical analyses

All analyses were performed using SPSS (ver 19.0, IBM, Armonk, NY, USA) and the figures were produced using Origin (ver 2022, OriginLab, USA). One‐way ANOVA was used to analyse the differences in the amount of DRs and the functional traits of *C. filispica* after different trampling treatments, if any, using LSD post hoc tests to indicate the significant difference between two treatments. Two‐way ANOVA was used to analyse the interaction between the presence of DRs and trampling intensity on each functional trait. Pearson correlation analysis was used to analyse the correlation between the amount of DRs and the functional traits of individuals with and without DRs. Principal component analysis (PCA) was used to downscale the standardized functional traits related to the economic spectrum of leaves and roots and calculate the score of their first axis respectively. General linear regression model (GLM) was used to simulate the correlation between the principal component scores of both the leaf and root economic spectrum of individuals with and without DRs after different treatments and their correlation with biomass.

## 3 RESULTS

### 3.1 Response of dauciform roots to trampling

**FIGURE 1:**
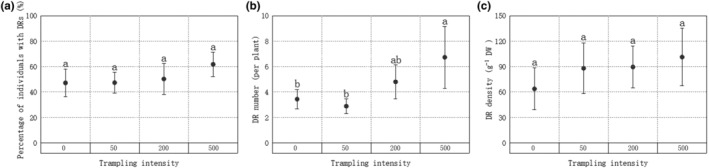
Differences in dauciform root (DR) traits among different trampling intensities in (a) the proportion of individuals with DRs; (b) number of DRs; and (c) density of DRs. Bars represent Means ± SE (standard errors). Different letters indicate a significant difference among trampling intensities (*p* < .05).

The mean number of dauciform roots differed significantly (*p* = .033, *F* = 3.038, *df* = 103) among different treatments (Figure 1b) while trampling intensity showed a positive correlation with the number of dauciform roots (*r* = .284, *p* = .004). Post hoc test indicated that the DR number after 500 passages was significantly higher than that of 50 passages and the control group without trampling (Figure 1b). Even though the proportion and density of DRs did not show any significant difference across treatments, they also had an increasing trend with the increasing trampling intensity, similar to the trend of DR number, as can be seen in Figure 1.

### 3.2 Response of functional traits to trampling

**FIGURE 2:**
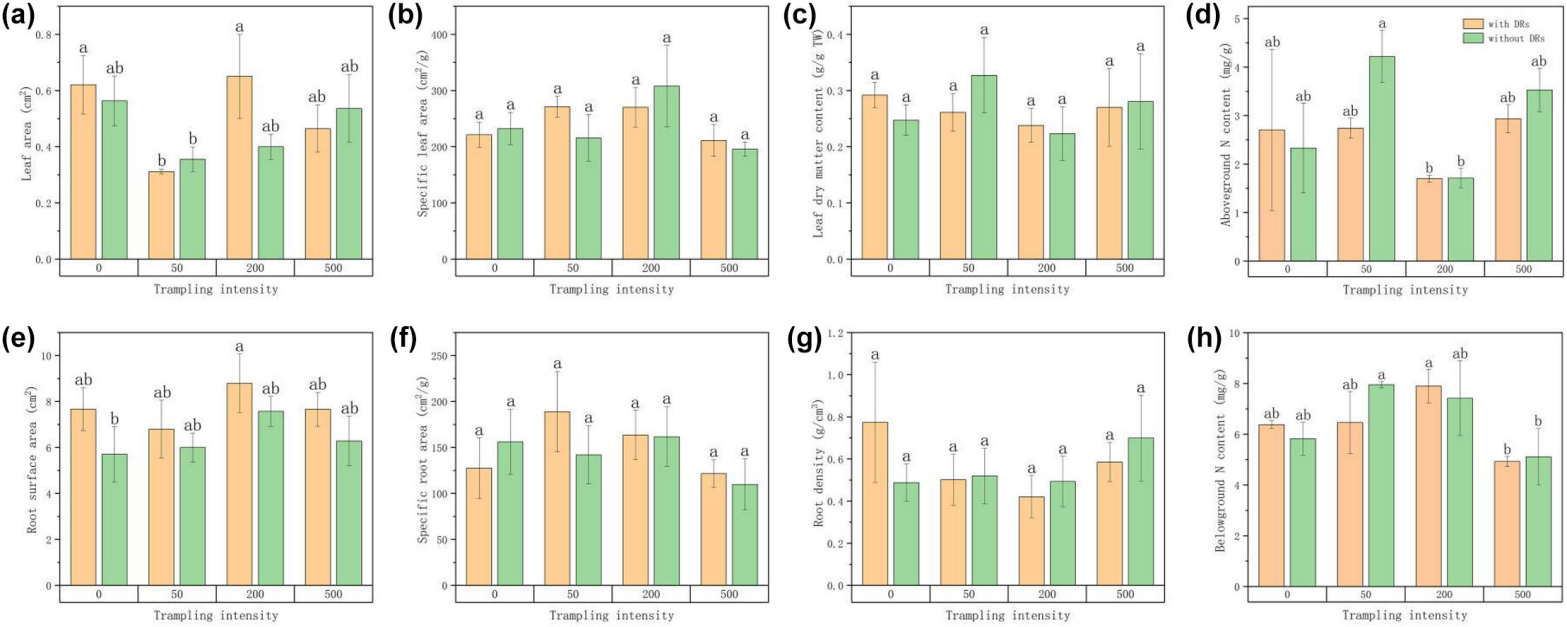
Differences in functional traits among different trampling intensities in (a) leaf area; (b) specific leaf area; (c) leaf dry matter content; (d) aboveground nitrogen (N) content; (e) root surface area; (f) specific root area; (g) root density; and (h) belowground nitrogen (N) content. Bars represent Means ± SE (standard errors). Different letters indicate a significant difference among trampling intensities (*p* < .05).

There was no significant difference in morphological traits between *C. filispica* with and without DRs at all trampling intensities (Figure 2). Moreover, there was no significant difference in morphological traits among treatments with different trampling intensities, except for the leaf area of individuals with DRs, which differed among 0, 50 and 200 passages. Both the belowground N content of individuals with and without DRs showed a parabolic trend, with higher belowground N content after recovering from low levels of trampling of 200 and 50 passages respectively.

Two‐way ANOVA (Table 1) showed that the trampling intensity had a significant effect on leaf area, consistent with Figure 2, while the interaction between the presence of DRs and trampling intensity was not significant on any trait, that is, there was no significant difference between the effects of trampling on individuals with and without DRs.

**TABLE 1:** A two‐way analysis of variance results for the effects of dauciform root (DR) presence, trampling intensity and their interaction on the functional traits.

	LA	SLA	LDMC	AGN	RA	SRA	RTD	BGN
Trampling	.030*	.175	.618	.145	.340	.425	.541	.058
DR presence	.441	.835	.905	.433	.069	.733	.848	.796
Interaction	.279	.649	.700	.629	.949	.692	.530	.626

Abbreviations: AGN, aboveground nitrogen content; BGN, belowground nitrogen content; LA, leaf area; LDMC, leaf dry matter content; RA, root surface area; RTD, root density; SLA, specific leaf area; SRA, specific root area.

* Indicates a significant difference (*p* < .05).

### 3.3 Relationship between dauciform roots and functional traits

**FIGURE 3:**
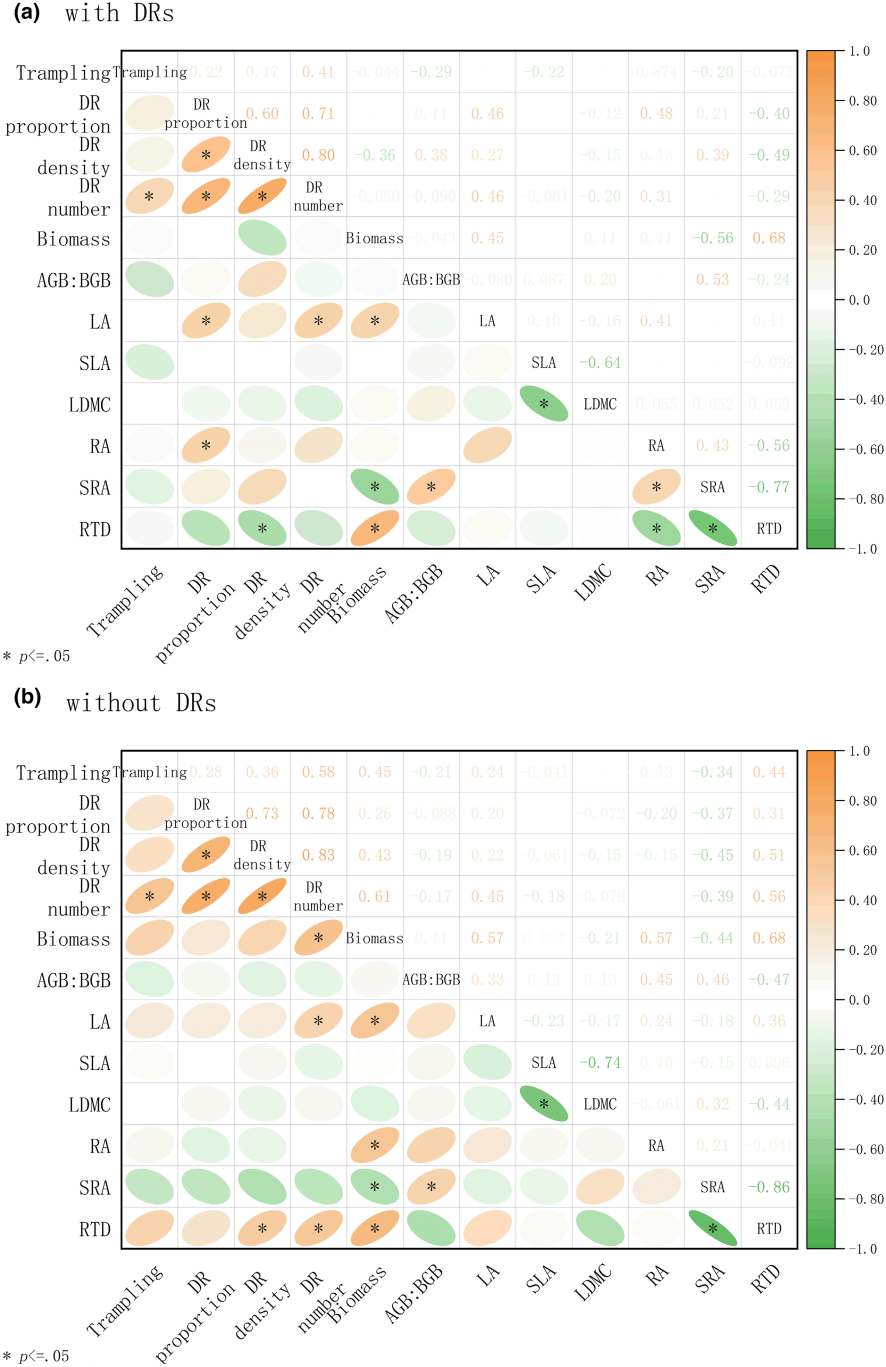
Relationships among trampling intensity, dauciform root (DR) traits and morphological traits of *Carex filispica*, described by Pearson correlation coefficients, separated by dauciform root (DR) presence. (a) Relationships of individuals with DRs. (b) Relationships of individuals without DRs. AGB, BGB, above‐ to belowground biomass ratio; LA, leaf area; LDMC, leaf dry matter content; RA, root surface area; RTD, root density; SLA, specific leaf area; SRA, specific root area. *Indicates a significant correlation (*p* < .05).

As consistent with Figure 1, trampling intensity showed a positive correlation with the number of DRs (Figure 3). The proportion of DRs was positively related to the leaf area and root area of individuals with DRs. The density of DRs was negatively related to the root density (RTD) of individuals with DRs but positively related to those without DRs. The number of DRs was positively proportional to both the leaf area of individuals with and without DRs, as well as to the biomass of those without DRs. The above‐ to belowground biomass ratios of both individuals with and without DR were inversely proportional to their specific root area (SRA).

### 3.4 Economic spectrum and trampling recovery

**FIGURE 4:**
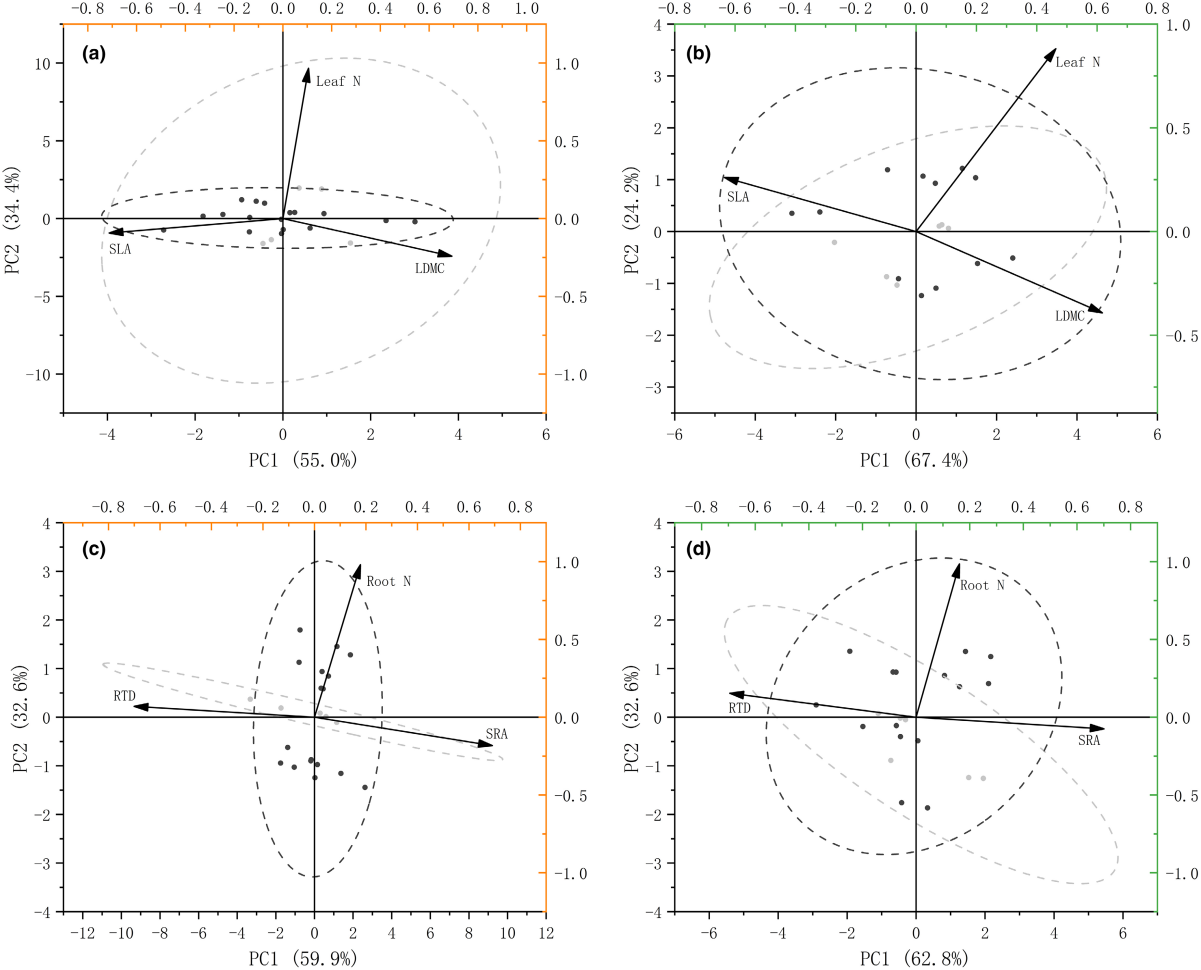
Principal component analysis (PCA) of (a) specific leaf area (SLA), leaf dry matter content (LDMC) and aboveground nitrogen (N) content of plants with DRs; (b) specific leaf area (SLA), leaf dry matter content (LDMC) and aboveground nitrogen (N) content of plants without DRs; (c) specific root area (SRA), root density (RTD) and belowground nitrogen (N) content of plants with DRs; (d) specific root area (SRA), root density (RTD) and belowground nitrogen (N) content of plants without DRs. The light‐grey ellipses indicate the 95% confidence intervals of the control group and the dark‐grey ones of trampling treatments.

Principal component analyses (PCA) were conducted separately for leaf economic spectrum traits and root economic spectrum traits of *C. filispica* after recovery from trampling. The first axis of the leaf economic spectrum was dominated by specific leaf area (SLA) and leaf dry matter content (LDMC), and the first axis of the root economic spectrum was dominated by specific root area (SRA) and root density (RTD). From Figure 4, it can be seen that the lower the SLA, which indicates a more resource‐conservative trend, the higher the value of principal component analysis axis 1 (PC1) of leaf traits, while the higher value of PC 1 of root traits indicates towards a more resource‐acquisitive direction. Further correlation analysis showed that the density of DRs was positively related to the value of PC 1 of root traits of individuals with DRs (*r* = .459, *p* = .031), indicating a co‐variation with the resource‐acquisitive root traits.

**FIGURE 5:**
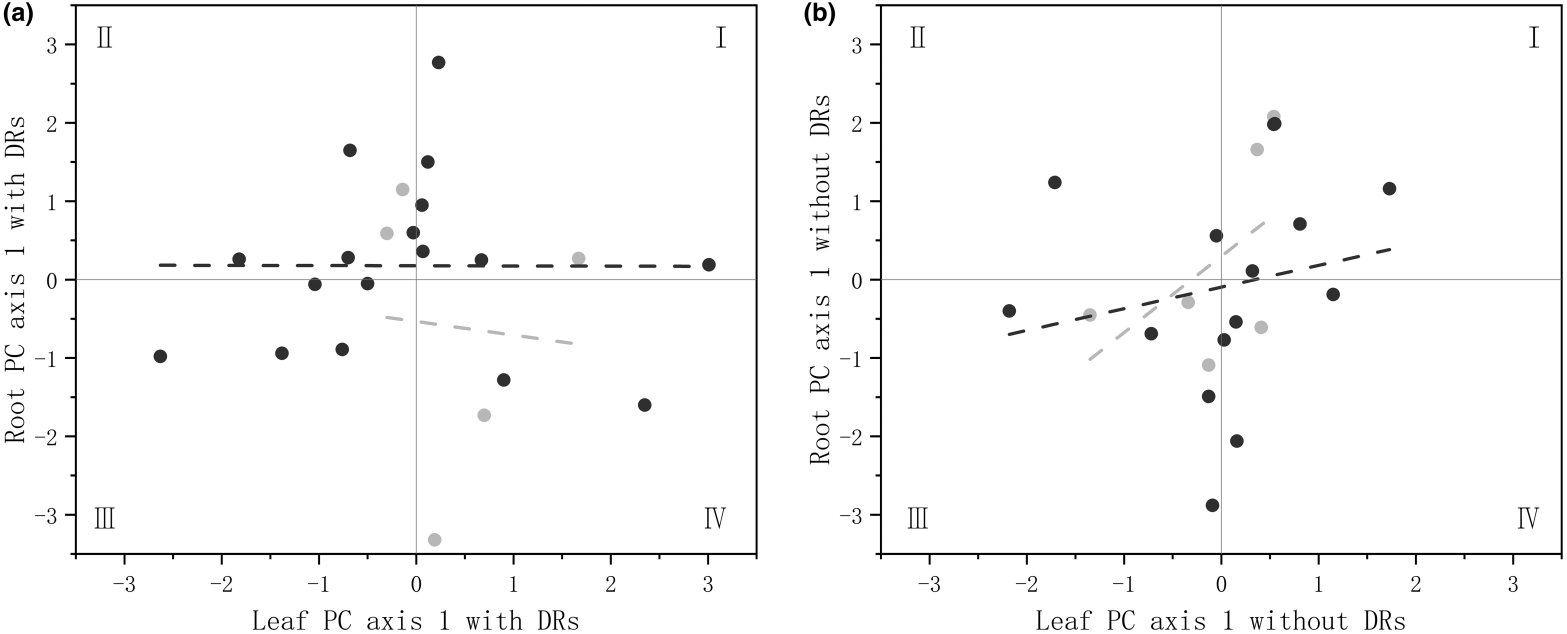
Relations between the economic spectra of leaf and root traits in (a) plants with DRs and (b) plants without DRs. The light‐grey line and dots indicate the control group and the dark‐grey ones indicate trampling treatments.

Therefore, in Figure 5, the first and second quadrants represent the root‐acquisitive direction, while the second and third quadrants represent the leaf‐acquisitive direction. The root PC 1 and leaf PC 1 of individuals with DRs showed no significant correlation, with the root generally biased towards the direction of resource acquisition (i.e. most points in the first two quadrants). The root PC 1 and leaf PC 1 of individuals without DRs were positively correlated, and were mostly distributed in the direction of the first and third quadrants, that is, leaf acquisition was positively correlated with root conservation.

**FIGURE 6:**
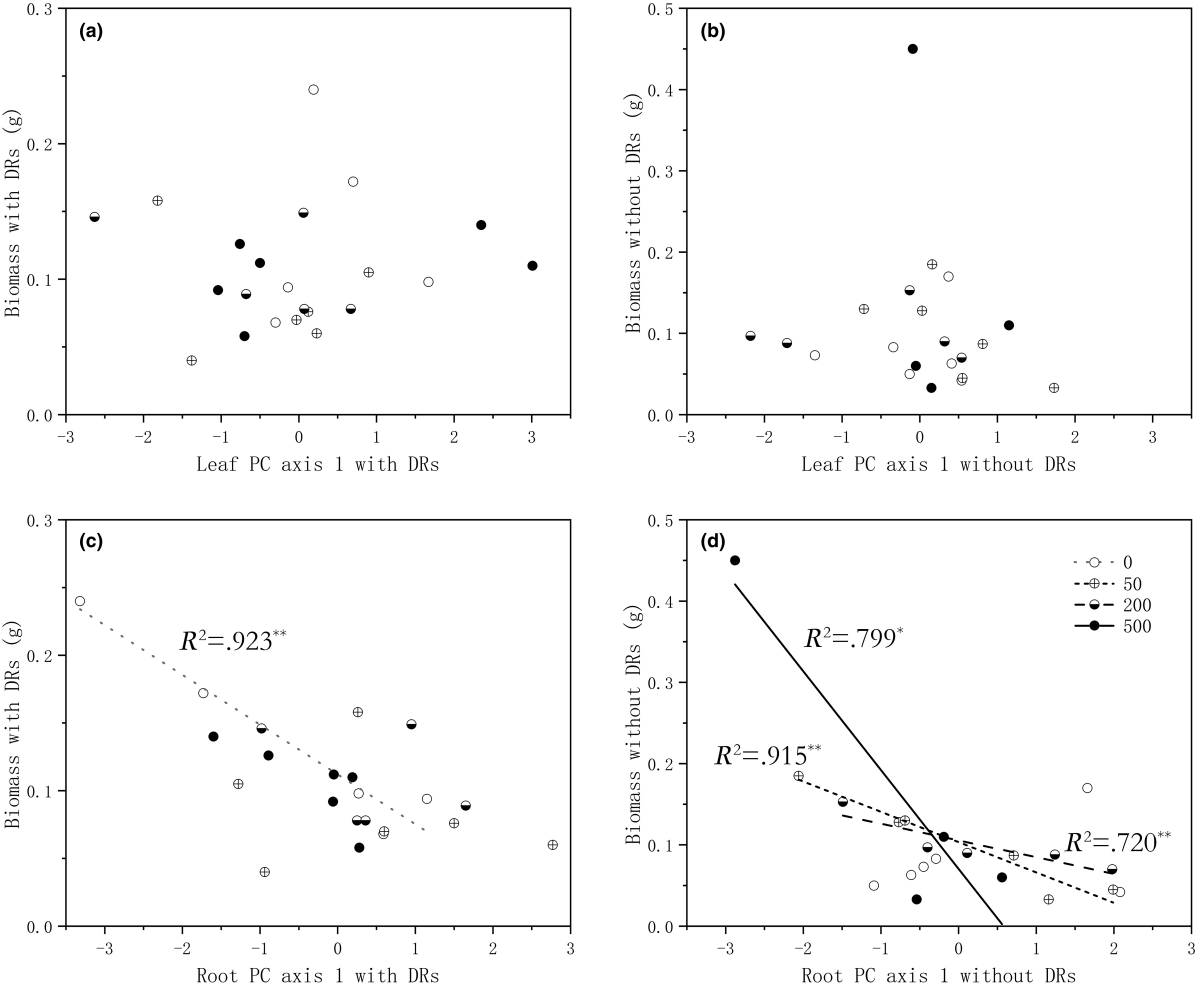
Relations of (a) the value of principal component analysis axis 1 (PC1) of leaf in individuals with dauciform roots (DRs) and their biomass; (b) the value of principal component analysis axis 1 (PC1) of leaf in individuals without dauciform roots (DRs) and their biomass; (c) the value of principal component analysis axis 1 (PC1) of root in individuals with dauciform roots (DRs) and their biomass; and (d) the value of principal component analysis axis 1 (PC1) of root in individuals without dauciform roots (DRs) and their biomass. *p < .05, **p < .01.

The biomass of individuals with and without DRs were both independent from their position on the leaf economic spectrum but inversely related to the PC 1 of the root economic spectrum, with the correlation coefficients of *r* = −.657, *p* = .001 for individuals with DRs and *r* = −.605, *p* = .004 for those without respectively. Plants with resource‐conservative roots showed better growth after trampling, which is consistent in both individuals with and without DRs. Interestingly, further analysis showed that individuals with DRs only showed a significant resource‐conservative advantage to biomass in the control group, while individuals without DRs showed only after trampling of different degrees (Figure 6).

## 4 DISCUSSION

### 4.1 Dauciform roots

Trampling leads to a shift in competitiveness from sensitive species to more tolerant ones (Miehe et al., 2019). With the increasing grazing disturbance, grasses and legumes on Tibetan plateau meadows are gradually replaced by sedge (Guo et al., 2019), which has been found to have a strong short‐term resistance to trampling, with no significant decrease in cover as the trampling intensity increases (Fan et al., 2023). As for this experiment, 1 year after trampling, the proportion of plants with DRs increased with trampling intensity, although the difference was not significant (Figure 1).

Previous studies have shown that 2 weeks after trampling, 50 passages led to a significantly higher proportion and density of DRs than the control group as well as the treatments of 200 and 500 passages (Fan et al., 2023). After minor trampling, the stimulation and changes in the microenvironment may promote root production, provide nutrients for aboveground regeneration and lead to compensatory growth (Jiang et al., 2021; Xu et al., 2014). Yet, in the present study, 1 year after trampling, the treatment with 500 passages had significantly more DRs than the control group, showing a significantly positive correlation between trampling intensity and the number of DRs, that is, the more severe the trampling, the more DRs. Therefore, our first hypothesis was confirmed: the long‐term response of dauciform roots to trampling showed a significant difference from the short‐term. The response of plants to trampling in the short term mostly reflects the resistance of their leaves to direct damage, whereas resilience in the long term reflects the ability of the root system to recover and regenerate (Liddle, 1975). The positive relationship between the number of DRs and trampling intensity 1 year later would suggest that the presence of DRs has a positive effect on root regeneration, which therefore makes them a more common trait in lanes with higher trampling intensity. On the other hand, previous studies have found that grazing disturbances often affect the diversity of plant communities by causing increased competition for available soil nutrients, especially phosphorus (Niu et al., 2016), and with DRs being considered as a response to low phosphorus conditions (Shane et al., 2005), this may also help to explain their increase.

### 4.2 Dauciform roots and functional traits

Compared to lower altitudes, alpine communities have less resistance and resilience to trampling (Ballantyne et al., 2014), and furthermore, wet habitats appeared to be extra sensitive (Francis et al., 2005; Liddle, 1975). However, in our experiments, 1 year after trampling, even though we did not directly quantify recovery, the economical spectrum traits (SLA, LDMC, Leaf N, SRA, RTD and Root N) of *Carex filispica* showed no significant difference from the control group and therefore appeared to be unaffected (Figure 2), indicating that they were able to recover from damage caused by trampling of different intensities.

While plants with small leaves are likely to be more resistant, larger leaf areas are often associated with faster growth thus recovery (Cornelissen et al., 2003), and plants tend to invest biomass in organs contributing to the capture of the most limited resources (Bloom et al., 1985; Poorter et al., 2012). Previous studies have found that sedges with DRs have relatively less belowground biomass allocation and suggested that DRs may ease the limitation of belowground resources so that resources can be invested in the growth of aboveground parts (Gusewell & Schroth, 2017). The results of the 2‐week recovery showed that the number of DRs was positively proportional to the overall leaf size of *C. filispica* (Fan et al., 2023), but since the morphology was not measured separately in individuals with or without DRs, the exact object of effect could not be determined. This study further found that both the leaf area of individuals with and without DRs showed a positive correlation with the number of DRs (Figure 3). The number of DRs had a positive relationship with the growth status (leaf area and biomass) of individuals without DRs, suggesting that DRs, with the ability to dissolve soil‐fixed phosphorus, may have a positive effect on the neighbouring plants as well. Previously, it was found that the leaf area and aboveground biomass of sedge were able to remain stable or even increase with the degradation of alpine meadows, while all other functional groups showed a significant decreasing trend (Hao et al., 2020; Ma et al., 2010). This phenomenon may be explained by that DRs could provide better resource utilization in disturbed environments, which could provide an additional advantage for the aboveground competition of sedge.

The above‐ to belowground biomass ratios of both individuals with and without DRs were positively related to the specific root area (Figure 3), indicating that the more resource acquisitive the roots tend to be, the more effective the belowground inputs are, and the more resources can be allocated to the aboveground parts. Previous studies found that plants without DRs had a smaller aboveground‐to‐belowground ratio and believed that their extra inputs to the belowground parts balanced out the effects of DRs, so the presence or absence of DRs did not show any significant advantages or disadvantages (Gusewell & Schroth, 2017). However, the above conclusions were drawn from species‐level‐based studies, and even though our study found that, intraspecifically, plants with DRs have a smaller (although insignificant) above‐to‐belowground ratio, showing a high investment in short‐lived roots (Freschet et al., 2015), there is no significant difference between functional traits of individuals with and without DRs in their response to trampling, which disagrees with our second hypothesis.

### 4.3 Dauciform roots and economic spectrum

The resistance to trampling after 2 weeks showed that DRs are associated with multiple resource‐acquisitive traits, with larger leaf areas and finer roots. However, after 1 year of recovery from trampling, only the root traits of plants with DRs showed a tendency towards resource acquisition, while leaf traits did not show any significant trend (Figure 5). There is a theory suggesting that the plasticity of belowground morphological traits in plants may be genetically limited (Kramer‐Walter & Laughlin, 2017) and therefore not as important as aboveground ones for resource acquisition (Freschet et al., 2015). However, the plasticity of economic spectrum traits varies considerably in the same species under different conditions (Li et al., 2016; Violle et al., 2007): in this study, roots were more plastic, and the plants' position on the root economic spectrum was significantly correlated with their growth status (Figure 6). Overall, individuals with a resource‐conservative position on the root economic spectrum had a higher total biomass: in particular, individuals with DRs only showed the advantage of resource‐conservative roots when they were not trampled, while those without DRs showed this advantage only after different intensities of trampling (Figure 6).

Plants with a tighter correlation between leaves and roots tend to be more adaptive to extreme environments (Freschet et al., 2015; Liu et al., 2010): the coordination can help the plants obtain limited resources or inhibit their needs (Du et al., 2019). Plants tend to achieve fast growth by resource‐acquisitive leaf traits (high SLA, low LDMC) or root traits (high SRA, low root diameter), while having both leads to faster growth (Simpson et al., 2020). However, in this study, there was no correlation between the root economic spectrum and leaf economic spectrum for individuals with DRs (Figure 5a), while root and leaf acquisition were negatively correlated for individuals without DRs, that is, with the root trait leaning towards resource conservation, the leaf traits tended to lean towards resource acquisition. Moreover, trampling enhanced the positive correlation between conservative roots and biomass increase in individuals without DRs, while individuals with DRs showed no such effect and the density of DRs was positively correlated with the resource acquisition of roots in individuals with DRs. This can be explained by that a lower SRA usually represents a more resource‐conservative root and appears with an increase in belowground biomass, while the presence of DRs increases the SRA, which is the opposite of the root economics dominated by fungi collaboration (Bergmann et al., 2020) yet benefited the growth of plants and weakened this correlation. Thus, it is possible that the increase in the density of DRs with increased trampling contributed to the aboveground inputs, so that the total biomass did not decrease with resource‐acquisitive roots, but rather maintained a balance.

The screening of functional traits by the environment is not only among species but also within species (Sandel et al., 2021). Roots are primarily responsible for nutrient and water uptake, while leaves are most critical for light competition (Rehling et al., 2021), although this relationship applies mainly to individually growing plants, which becomes complicated as the neighbouring plants compete for the same resources (Donald, 1958; Dybzinski et al., 2011). In contrast, sometimes at high density, plants reduce their resource‐obtaining traits, which not only can be explained by that the high density restricts the growth of each individual (Rehling et al., 2021) but has also been explained by a mechanism to avoid intraspecific competition (Bennett et al., 2016; Novoplansky, 2009). In our study, individuals with DRs had resource‐acquisitive roots with an increasing density of DRs, which invested in the uptake of nutrients. Nevertheless, the biomass of plants was positively correlated with resource‐conserving roots, regardless of the presence or absence of DRs. Meanwhile, we learned that the number of DRs had a positive relation with leaf area and biomass of individuals without DRs. Thus, there is a possibility that the presence of DRs promotes the growth of the species as a whole: at the cost of the disadvantage of resource‐acquisitive roots in individuals that produce DRs, sedge ensures the overall growth and therefore plays a dominant role in alpine meadows. However, whether DRs have a beneficial effect on the growth of neighbouring plants requires further laboratory‐based controlled studies.

## 5 CONCLUSION

In this study, we found that the number of dauciform roots increased with trampling intensity after a long‐term recovery, which has a positive relationship with the aboveground growth of both individuals with and without DRs. Moreover, the presence of DRs may be related to the plants' position on the economic spectrum, thus influencing the resilience of *C. filispica* to trampling. In conclusion, this study contributes to our further understanding of the response of DRs to trampling and their effect on the functional traits of plants, building a foundation for future research on DRs; on the other hand, this study found that *C. filispica* showed strong recovery from trampling after 1 year, which makes them an adequate species for ecological restoration in alpine meadows, providing a future reference.

## AUTHOR CONTRIBUTIONS

**Rong Fan:** Conceptualization (equal); formal analysis (lead); investigation (equal); writing – original draft (lead). **Wanting Liu:** Investigation (equal). **Songlin Jiang:** Investigation (equal). **Yulin Huang:** Investigation (equal). **Wenli Ji:** Conceptualization (equal); funding acquisition (lead); supervision (lead).

## CONFLICT OF INTEREST STATEMENT

This manuscript is approved by all authors for publication with no conflict of interest. The work described was original research that has not been published previously, or is it under consideration for publication elsewhere.

## Data Availability

Fan, Rong et al. (Forthcoming 2023). Recovering from trampling: The role of dauciform roots to functional traits response of *Carex filispica* in alpine meadow [Dataset]. Dryad. https://doi.org/10.5061/dryad.3bk3j9kq0.
